# Inactivation of *Streptococcus mutans* genes *lytST* and *dltAD* impairs its pathogenicity *in vivo*

**DOI:** 10.1080/20002297.2019.1607505

**Published:** 2019-05-09

**Authors:** Midian C. Castillo Pedraza, Pedro L. Rosalen, Aline Rogéria Freire de Castilho, Irlan de Almeida Freires, Luana de Sales Leite, Roberta C. Faustoferri, Robert G. Quivey Jr, Marlise I. Klein

**Affiliations:** a Department of Dental Materials and Prosthodontics, Sao Paulo State University (Unesp), School of Dentistry, Araraquara, Brazil; b Department of Physiological Sciences, Piracicaba Dental School, University of Campinas – UNICAMP, Piracicaba, Brazil; c Department of Pediatric Dentistry, Piracicaba Dental School, University of Campinas – UNICAMP, Piracicaba, Brazil; d Center for Oral Biology, University of Rochester, Rochester, NY, USA

**Keywords:** Exopolysaccharides, eDNA, lipoteichoic acids, dental caries, systemic infection, oxidative stress

## Abstract

**Background: ***Streptococcus mutans* orchestrates the development of a biofilm that causes dental caries in the presence of dietary sucrose, and, in the bloodstream, *S. mutans* can cause systemic infections. The development of a cariogenic biofilm is dependent on the formation of an extracellular matrix rich in exopolysaccharides, which contains extracellular DNA (eDNA) and lipoteichoic acids (LTAs). While the exopolysaccharides are virulence markers, the involvement of genes linked to eDNA and LTAs metabolism in the pathogenicity of *S. mutans* remains unclear. **Objective and Design: **In this study, a parental strain *S. mutans* UA159 and derivative strains carrying single gene deletions were used to investigate the role of eDNA (Δ*lytS* and Δ*lytT*), LTA (Δ*dltA* and Δ*dltD*), and insoluble exopolysaccharides (Δ*gtfB*) in virulence in a rodent model of dental caries (rats) and a systemic infection model (*Galleria mellonella* larvae). **Results: **Fewer carious lesions were observed on smooth and sulcal surfaces of enamel and dentin of the rats infected with ∆*lytS*, ∆*dltD*, and Δ*gtfB* (vs. the parental strain). Moreover, strains carrying gene deletions prevented the killing of larvae (vs. the parental strain). **Conclusions: **Altogether, these findings indicate that inactivation of *lytST* and *dltAD* impaired *S. mutans* cariogenicity and virulence *in vivo*.

*Streptococcus mutans* is considered a primary agent for dental caries development since it metabolizes dietary sugars into organic acids and produces exopolysaccharides, which trap acids in microniches within biofilms and at the interface between biofilms and tooth surfaces, causing tooth demineralization [1,2]. In addition, *S. mutans* can also cause systemic infections when it reaches the bloodstream (bacteremia) and persist in tissues such as the heart (endocarditis) [3–5]. In both cases, *S. mutans* uses its ability to form biofilms and to withstand environmental stresses (oxidative, nutritional, among others). Biofilms are communities of microbial cells structured and organized in a dynamic environment, covered and embedded within a three-dimensional (3D) extracellular matrix [6,7]. The matrix of cariogenic biofilms formed by *S. mutans* is rich in exopolysaccharides and contain extracellular DNA (eDNA) and lipoteichoic acids (LTAs) that interact with exopolysaccharides [8]. While exopolysaccharides are proven cariogenic factors [2], it remains to be determined how genes associated with eDNA and LTA metabolism affect the occurrence of dental caries and systemic infection.

The presence of carbohydrates (sucrose and starch) contributes to the formation of the matrix in cariogenic biofilms. The matrix provides stability and structural integrity by allowing bacteria to adhere to tooth surfaces and provide protection against harmful environmental stimuli or other attacks [9]. The adhesion mechanism is guided by the glucosyltransferase (Gtfs) exoenzymes, which synthesize exopolysaccharides [9]. Furthermore, when carbohydrates are present in the oral cavity, acid production in the biofilm increases [10]. Organic acids can be retained in low pH niches within the biofilm because the exopolysaccharide-rich matrix prevents neutralization of these acids by saliva [11]. *S. mutans* encodes three different Gtfs, namely: GtfB, which produces water-insoluble exopolysaccharides (glucans); GtfC, which produces both water-soluble and -insoluble exopolysaccharides (glucans); and GtfD, which produces soluble exopolysaccharides (glucans); these enzymes are also known as Gtf-I, Gtf-SI and Gtf-S, respectively [9].

In addition to exopolysaccharide production, *S. mutans* can release eDNA and LTA that will interact with exopolysaccharides [8,12]. Sucrose and starch induce the expression of *gtfB* [13,14], the two-component system *lytST* (associated with cell lysis and cell-wall remodeling) [13,14], and the operon *dltABCD* (related to LTA metabolism) [13,15]. This augmented expression increases the abundance of exopolysaccharides, eDNA and LTA that enable the construction of a bulky and sturdy extracellular matrix in *S. mutans* biofilms [8,14,16].

eDNA can be released during cell lysis and via microvesicles [17]. Reduction in *lytST* gene expression results in a decrease in eDNA presence in the matrix during the biofilm development process [14]. LTA is a cell wall polymer of Gram-positive bacteria, consisting of 1,3-polyglycerol-phosphate bound to the cell membrane by a lipoprotein, which during the remodeling of the cell wall or cell division, can be released into the extracellular environment [18,19]. LTA plays a significant role in the colonization of Gram-positive bacteria, contributing to the formation and pathogenicity of biofilms, and is associated with adherence and colonization of oral streptococci [20,21]. D-alanine promotes adhesion and biofilm development [22,23] and the *dltABCD* (D-alanyl-LTA) genes metabolize these residues during LTA synthesis [24]. Of note, activation of the *dltA* and *dltD* genes occurs during the early stages of matrix formation in mixed-species cariogenic biofilms [15].

In an *in vitro* study the *S. mutans* deletion strains ∆*lytS*, ∆*lytT*, ∆*dltA*, and ∆*dltD* contributed to an increased amount of extracellular eDNA and LTA in the matrix of biofilms, compared to either the parental *S. mutans* strain, UA159, or the ∆*gtfB* strain [8]. The presence of eDNA and LTA in the matrix resulted in an increased amount of soluble and insoluble exopolysaccharides, indicating that these biofilms could be more cariogenic (with a potential to cause increased caries) [8]. Thus, the evaluation of these genes associated with the essential components of the matrix (exopolysaccharides, eDNA and LTA) may help to clarify their contribution to the pathogenicity of *S. mutans*. Moreover, it is still unknown how these deletion strains behave when subjected to a systemic infection model. Thus, it is essential to clarify the functions of *lytST* and *dlt* genes to better understand the biology of *S. mutans* and to direct therapies to control biofilm formation by this species. The purpose of this research was to analyze the virulence of the *lytST* and *dltAD* genes of *S. mutans* in the development and severity of carious lesions (in a rodent model of dental caries) and virulence in a systemic infection model (*Galleria mellonella*).

## Materials and methods

### Bacterial strains

*Streptococcus mutans* UA159, serotype *c* (ATCC 700,610), a proven cariogenic organism, was the parental strain used in this study. Mutant strain derivatives of UA159 were created carrying deletions in *lytS, lytT, dltA* and *dltD*. The deletion strains for *lytS, lytT* [25], *dltA*, and *dltD* [26] came from the collection of mutant strains created in the Quivey laboratory (kindly provided by Dr. Robert G. Quivey Jr, Center for Oral Biology, University of Rochester, Rochester, NY). In addition, a strain defective in *gtfB* was used as a control, because it displays impaired biofilm formation *in vitro* [11,27], and reduced pathogenicity *in vivo* [28]. The *gtfB* mutant was kindly provided by Dr. Robert A. Burne (Department of Oral Biology, University of Florida, Gainesville, FL). The strains were stored at – 80°C in tryptic soy broth containing 20% glycerol and were plated on blood agar plates or BHI agar plates with recommended antibiotic before use in the following assays.

### Rat model of dental caries

**Figure 1.**
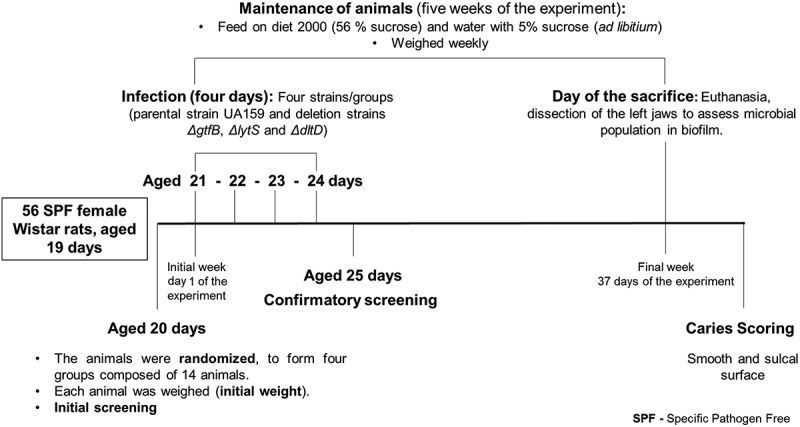
Experimental design for the rat model of dental caries.

The animal experiment was reviewed and approved by the Ethical Committee on Animal Research at the University of Campinas (CEUA – Ethics Committee on Animal Use/UNICAMP, Campinas, SP, Brazil, process number 4463–1/2017) and was performed according to methods previously described [29,30]. An overview of the experimental design is shown in Figure 1. A total of 56 SPF (Specific Pathogen Free) female Wistar rats, aged 19 days were provided by CEMIB/UNICAMP (Multidisciplinary Center for Biological Research, Campinas, SP, Brazil). From the arrival of the animals until the end of the experiment, the animals were kept in a room of the vivarium for research using Genetically Modified Organisms (GMOs), authorized and approved by the GMO Biosafety Committee from FOP/UNICAMP (A001-2017).

On the day after the arrival of the animals, they were randomized to form four groups composed of 14 animals, using ear tags. The four groups were color-coded, so the investigators handling the animals were blinded to the strain that would be used for infection. The black group was infected with the parental strain, UA159 (positive control strain). The red group was infected with Δ*gtfB* (a reduced-caries-causing control to which the caries-causing potential of the tested strains was compared). The blue and green groups were infected with Δ*lytS* and Δ*dltD*, respectively.

Each animal was weighed (COMTEC 426–1070) and the data recorded. Initial screening was performed to verify that the animals were not colonized with mutans streptococci. A sterile cotton bud (Labor Import), wetted in saline solution (0.89% NaCl), was used to swab the rat mouths. Each oral swab was struck on a Mitis Salivarius agar plate without antibiotic (MSA) and on a Mitis Salivarius agar plate containing 0.2 U of bacitracin per ml (MSB). The plates were incubated (37°C, 95% (v/v) air/5% (v/v) CO_2_, 48 h) and the morphologies of colonies were visually evaluated to ensure that the animals were free of mutans streptococci colonization.

#### Biofilm and inocula preparation for caries animal study

The four strains (parental strain UA159 and deletion strains *ΔgtfB, ΔlytS*, and *ΔdltD*) were grown in biofilm cultures on glass rods in the presence of sucrose. It has been shown that using biofilm cultures as the inoculum in the rat mouth enhances their ability to colonize tooth surfaces [31,32]. On day one of biofilm growth, 50 µL of each strain was transferred to a tube containing TY (2.5% tryptone plus 1.5% yeast extract, Difco) and 2% sucrose (w/v) (Dinamica®/1894–1), antibiotic was added to media for cultures of the deletion strains. After incubation at 37°C in a 95% (v/v) air/5% (v/v) CO_2_ atmosphere for 24 h (day two of biofilm growth), each rod was transferred to new tubes with fresh media containing 2% sucrose. These cultures were incubated under the same conditions described above, and the rods transferred to fresh media again on day three of biofilm growth. On day four of biofilm growth, biofilms were scraped off the rods and transferred to a new tube containing fresh media, then incubated as above. After 18 h, these cultures were plated on MSB agar plates and incubated for 48 h, after which time the plates were stored at 4°C to be utilized as inocula to infect the animals.

On the day before the arrival of the animals, starter cultures were prepared by inoculating 10 mL of TY liquid medium containing 1% glucose (w/v; Synth) with colonies from the plates described above. Two tubes were prepared for each strain and incubated at 37°C in a 95% air/5% CO_2_ atmosphere. After 18 h, the starter cultures were diluted in TY liquid medium containing 1% glucose (1:20 dilution). The diluted cultures were allowed to grow to an O.D._540nm_ ~ 0.5. Each culture was then transferred to a 50 mL tube containing 14 swabs. These tubes were maintained on ice until shortly before inoculating the animals' mouths, at which time the cultures were warmed to room temperature.

#### Infection and maintenance of animals

Rats were infected with the cultures described above by oral swabbing four times, on four consecutive days (as outlined in Figure 1). After these four infection procedures, a confirmatory screening was performed to verify the presence of the *S. mutans* strains in the mouth of each rat, similar to the procedure used in the initial screening. Immediately after the confirmatory screening, the animals were transferred to suspended cages (two animals per cage), where they remained for five weeks until sacrificed.

After the first infection, the rats’ diet was replaced with Diet 2000 (containing 56% sucrose [Table S1; 33]). The animals were given sterile, distilled, deionized, water containing 5% sucrose, ad libitum. The ad libitum cariogenic diet used during the five-week experimental period is an established method for allowing the development of carious lesions on the dental surfaces [9]. The animals were weighed weekly (Figure S1). In the second and fourth weeks, the vitamin Vitagold Potenciado (Fabiani Saúde Animal, São Paulo, Brazil) was added to the drinking water (20 mL per 10 L of water) to prevent hair loss.

#### Recovery of jaws from animals

The animals were euthanized by anesthesia using Ketamine (Dopalen 1,000mg/10 mL – Ceva ®) (300 mg/kg) and Xylazine (Anasedan 2,000 mg/10 mL – Ceva ®) (30 mg/kg), followed by decapitation.

The lower left jaw of each animal was aseptically dissected and was suspended in 5 mL of sterile 0.89% NaCl solution. The jaws were sonicated twice, with a 10-s interval between probe sonication for 30 s at 7 W (Vibracell, Sonics and Material Inc., Newtown, CT). Aliquots of each biofilm suspension were used for 10-fold serial dilutions. The dilutions 1:10, 1:100 and 1:1,000 and undiluted suspension were seeded on blood agar medium (for total cultured microbiota) and MSB agar medium (for *S. mutans*), and the plates were incubated for 48 h at 37°C in a 95% (v/v) air/5% (v/v) CO_2_ atmosphere. The blood agar plates were incubated for another 24 h at 37°C. Colonies were counted and used to calculate CFU in 5 mL of biofilm suspension (CFU/biofilm). All jaws were stored at −20°C until manual dissection for caries scoring.

#### Caries scoring

The teeth were prepared for caries scoring according to Larson’s modification of Keyes system [34]. One calibrated examiner performed the caries score of the codified jaws. The smooth surface caries scoring (buccal, morsal, proximal, lingual and palatinal) was done using a stereoscopic Zeiss magnifying lens (Stemi SV 6).

Before performing caries scoring on sulcal surfaces, the samples were prepared as follows: The jaws were painted with transparent nail polish to strengthen the structure and left to dry for 24 h. Murexide (SIGMA M-2628) dye solution (0.24 mg/mL) was used to stain the jaw and left to dry for 18 h. A sagittal cut was made in the jaws with a carbide disk coupled to a micromotor (mc52 – dent) to allow for better visualization of the sulcal surface. Caries scoring was performed on the sulcal surfaces using a stereoscopic Zeiss magnifying lens (Stemi SV 6).

### Infection study in the invertebrate model *G. mellonella*

Five deletion strains (∆*gtfB*, ∆*lytT*, ∆*lytS*, ∆*dltA* and ∆*dltD*) and the parental strain (UA159) of *S. mutans* were used to examine pathogenicity in the *G. mellonella* model of infection. The strains were cultured as described in ***Bacterial Strains***. Starter cultures of each of the six strains were prepared in 10 mL of TY liquid medium containing 1% glucose (w/v) and incubated at 37°C in a 95% (v/v) air/5% (v/v) CO_2_ atmosphere. After 18 h, the cultures were diluted in TY liquid medium containing 1% glucose (1:20 dilution) and grown to an O.D._540nm_ ~ 0.5.

Cells were collected by centrifugation (6,500 x *g*/4°C/15 min, Hettich Zentrifugen, rutin 420R) and pellets were washed twice with 3 mL of sterile 0.89% NaCl solution. Cell pellets were resuspended in 3 mL 0.89% NaCl. Briefly, a 100 μL aliquot of each strain suspension was transferred to a tube containing 900 μL of 0.89% NaCl solution to perform 10-fold serial dilutions. The 10^–7^ dilution was plated on blood agar (in triplicate), and the plates were incubated at 37°C in a 95% (v/v) air/5% (v/v) CO_2_ atmosphere. After 48 h, the colonies were counted and used for the calculation of CFU. A heat-killed control for each strain was incubated for 40 min at 85°C.

The systemic infection of *G. mellonella* was performed as described previously [35–37]. The larvae were stored at 32°C, in the dark, in a greenhouse (SOLAB BODSL-200/364, Solab, Piracicaba, Brazil) until the experiment. For each bacterial strain tested, a group of 10 larvae, varying from 200 to 300 mg and without signs of melanization, were selected for inoculation with the six strains of *S. mutans* (parental UA159, Δ*gtfB*, Δ*lytT*, Δ*lytS*, Δ*dltA* and Δ*dltD*) and the heat-inactivated control strains (all infections were performed in triplicate).

Five μL of *S. mutans* from each strain containing 1–1.5 × 10^8^ CFU was injected intra-hemocoel via the last left proleg using a Hamilton syringe (Hamilton, Reno NV) [38]. After injection, the larvae were placed in Petri dishes for incubation at 37°C in a 95% (v/v) air/5% (v/v) CO_2_ atmosphere. The appearance of melanization and larval survival were recorded three times per day according to the *G. mellonella* Health Index Scoring System [39]. The larvae were classified as dead when they displayed no movement in response to touch.

### Tolerance to oxidative stress by hydrogen peroxide (H_2_O_2_)

Planktonic cultures and biofilms of the six strains of *S. mutans* (parental UA159, Δ*gtfB*, Δl*ytT*, Δ*lytS*, Δ*dltA* and Δ*dltD*) were subjected to an H_2_O_2_ challenge, as oxidative stress is part of the innate immune system of *G. mellonella* [38,40]. The planktonic cultures were grown as described above for the infection of larvae. Cultures were exposed to 0.2% H_2_O_2_ for 0, 5, 15, 30 and 45 min. At the end of the exposure time, aliquots (100 µL) were used for 10-fold serial dilutions that were plated on blood agar plates.

The biofilm cultures were grown on vertically placed hydroxyapatite disks (1.25 cm diameter, Chromatography products Clarkson, Inc., South Williamsport, PA), coated with filter-sterilized clarified human whole saliva (sHA). The saliva and pellicle preparation were performed as described before [8]. The saliva collection and use were approved by the Institutional Ethics Committee on Human Research at UNESP (CAAE: 31717914.3.0000.5416). Biofilm formation was performed as described by Castillo Pedraza et al. [8]. Briefly, the strains were grown in TY broth containing 1% glucose (w/v) at 37°C in a 95% (v/v) air/5% (v/v) CO_2_ atmosphere until reaching late exponential growth phase (OD_540nm_ ~1.0).

Next, each strain was inoculated individually (10^6^ CFU mL^−1^) in 2.8 mL of TY with 0.1% sucrose (w/v) and 25% saliva (v/v) and incubated at 37°C in a 95% (v/v) air/5% (v/v) CO_2_ atmosphere. The beginning of this incubation was considered day 0 (biofilm age 0 h). At 19 h, the culture medium was replaced by transferring the custom-made disk holder with nascent biofilms to wells containing fresh medium (TY + 0.1% sucrose and 25% saliva) using forceps. After 29 h of biofilm growth, biofilms were transferred to TY containing 0.5% sucrose, (w/v) 1% starch (w/v), and 25% saliva to increase the amount of carbohydrate serving as substrates for microbial metabolism and matrix accumulation. The culture medium was then changed twice daily (8 am and 6 pm) until the end of the experimental period. Thus, the biofilms were kept immersed in 0.1% sucrose/25% saliva for 10 h, and in 0.5% sucrose/1% starch/25% saliva for 14 h. Biofilms were grown up to 67 h, and during all ages were incubated with the same initial environment as 0 h (at 37°C in a 95% (v/v) air/5% (v/v) CO_2_ atmosphere).

At 67 h biofilms were removed from the culture medium, rinsed (three times) with 0.89% NaCl solution, and transferred to wells of a 24-well plate containing 0.2% H_2_O_2_ and incubated for 15, 30 and 45 min. At time 0 min, biofilms were incubated without H_2_O_2_. At the end of the exposure time, the disks were transferred to tubes containing 2 mL 0.89% NaCl solution and sonicated for 10 min (in an ultrasonic bath) to harvest biofilms from the disks. The disks were scraped with a sterile spatula to remove residual biofilm. The biofilms were homogenized with a probe for 30 s at 7 W (Sonicator model Q125, QSonica). From these biofilm suspensions, aliquots (100 µL) were used for 10-fold serial dilutions that were plated on blood agar plates.

For both planktonic and biofilm cultures, the plates were incubated for 48 h at 37°C in a 95% (v/v) air/5% (v/v) CO_2_ atmosphere and the colonies were counted to obtain CFU/mL (planktonic cultures) or CFU/biofilm (biofilms). The population recovered at 0 min was set at 100% per strain for planktonic and biofilm cultures.

### Statistical analyses

The virulence potential of the different deletion strains and the parental strain of *S. mutans* were analyzed using Prism 7 software (GraphPad Software, Inc.). The microbial population data (total culturable microbiota, *S. mutans*, and percentage of *S. mutans*) recovered from rats were evaluated with analysis of variance (ANOVA) one-way followed by Tukey’s test (α = 0.05). The development of carious lesions was evaluated by two-way ANOVA, followed by the Tukey’s test (α = 0.05) using as factors ‘*S. mutans* strain’ and ‘type of caries lesion on smooth and sulcal surfaces’. The amount of CFU inoculated per strains of *S. mutans* in *G. mellonella* larvae was analyzed by one-way ANOVA, followed by the Tukey’s test (α = 0.05). The virulence potential of *S. mutans* in the invertebrate model was assessed using the Kaplan-Meier survival curve, and estimates of survival differences were compared using the log-rank test. Oxidative stress tolerance (H_2_O_2_) was evaluated using ANOVA two-way followed by Tukey’s test (α = 0.05) using as factors ‘*S. mutans* strain’ and ‘exposure time’ for both planktonic cultures and biofilms.

## Results

### Loss of lytS and dltD reduced caries formation in a rat model

**Figure 2.**
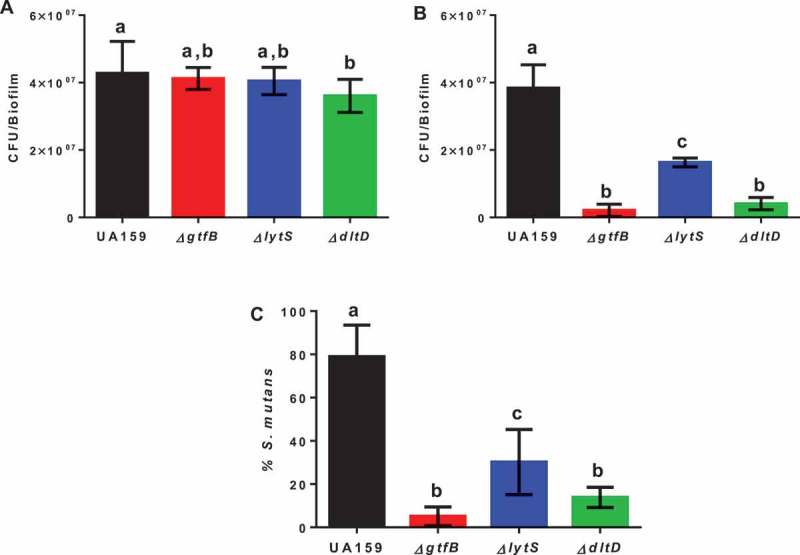
Microbial population recovered from the animals at the end of the caries study. The total cultivable microbiota (a) and *S. mutans* strains (b) derived from rats infected by parental strain UA159 and deletion strains Δ*gtfB*, Δ*lytS* and Δ*dltD*. (c) The percentage of *S. mutans* (%) in the total cultivable microbiota. Bars with the same letters indicate no statistical difference between the different strains in each graph (p > 0.05; one-way ANOVA, followed by Tukey’s test). The data represented are the means, and the error bars correspond to the standard deviation (n = 14 per group).

Total cultivable microbiota recovered from rats used in a model for caries formation revealed that rats infected with the parental strain UA159 and the Δ*gtfB* and Δ*lytS* strains, did not show significant differences between them, but the Δ*dltD* strain had fewer CFU/mL, compared to the parental strain UA159 (p = 0.0224, one-way ANOVA, followed by the Tukey’s test; Figure 2(a)). Infection with the Δ*gtfB* strain, our reduced caries control, resulted in a lower *S. mutans* population, compared to the other strains tested (parental strain UA159 and Δ*lytS* strain) except for the Δ*dltD* strain (no significant difference between ∆*gtfB* and ∆*dltD*; Figure 2(b)). The proportion of *S. mutans* in the total microbiota was higher for the parental strain UA159 compared to all deletion strains tested (p < 0.0001, one-way ANOVA, followed by the Tukey’s test; Figure 2(c)). Moreover, the percentage of *S. mutans* for Δ*gtfB* was lower than for Δ*lytS* (p = 0.0027), and there was no difference between Δ*gtfB* and Δ*dltD* strains (p = 0.2036). Therefore, there was a successful implantation of *S. mutans* in the oral microbiota of all rats evaluated.

**Figure 3.**
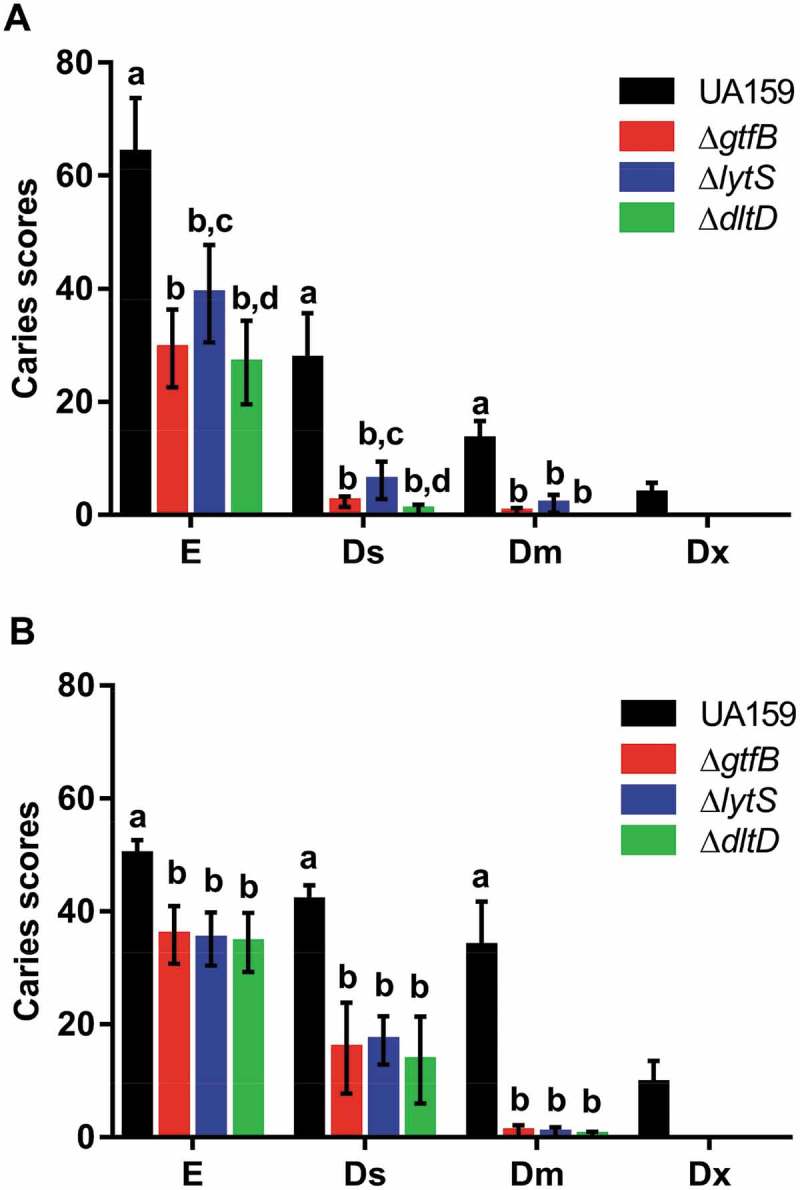
Influence of *S. mutans* strains on development and severity of dental caries on smooth (a) and sulcal (b) surfaces. The data displayed are the means, and the error bars correspond to the standard deviation (n = 14 per group). Bars with the same letters, by type of caries lesion, indicate no statistically significant differences between the strains (p > 0.05; two-way ANOVA, followed by the Tukey’s test). In the Dx category for both smooth and sulcal caries, no bars, other than *S. mutans* UA159, are represented, since the three deletion strains *ΔgtfB, ΔlytS* and *ΔdltD* did not yield this type of lesion. E: Enamel; Ds: light dentin caries; Dm: moderate dentin caries; and Dx: severe dentinal caries.

All strains tested caused dental caries on smooth and sulcal surfaces (Figure 3). There were significant changes in smooth carious lesion scores for rats infected with the deletion strains, compared to UA159 (Figure 3(a)); however, there was not a significant difference among the mutant strains. Animals infected with the *ΔlytS* strain exhibited a greater number of smooth surface carious lesions on enamel (E) and dentin (Ds and Dm) than rats infected with *ΔgtfB* and *ΔdltD* (Figure 3(a)). None of the mutant strains tested caused severe dentinal caries (Dx) on smooth and sulcal surfaces, though the rats infected with the Δ*lytS* strain displayed a significantly higher number of slight dentinal caries (Ds) on smooth surfaces than the Δ*dltD* strain (Figure 3(a)). These data suggest that *lytS* and *dltD* could play an important role in the development of caries on smooth surfaces. This observation has already been recorded for smooth surface lesions in animals infected with a ∆*gtfB* strain [28].

Sulcal carious lesions were reduced in rats infected with mutant strains, compared to infection with UA159. Moreover, there was no difference between the mutant strains in severity, but their scores were lower than those from UA159 infected animals (Figure 3(b)). Importantly, animals infected with the deletion strains also did not display Dx lesions on sulcal surfaces, suggesting that the products of the *lytS, dltD* and *gtfB* genes could also play an essential role in the development of caries on sulcal surfaces. Further, the loss of *lytS, dltD* and *gtfB* not only influenced the development of cariogenic biofilms *in vitro* [8] but also *in vivo*.

### Strains carrying gene deletions gtfB, lytT, lytS, dltA, and dltD prevented the killing of G. mellonella larvae

**Figure 4.**
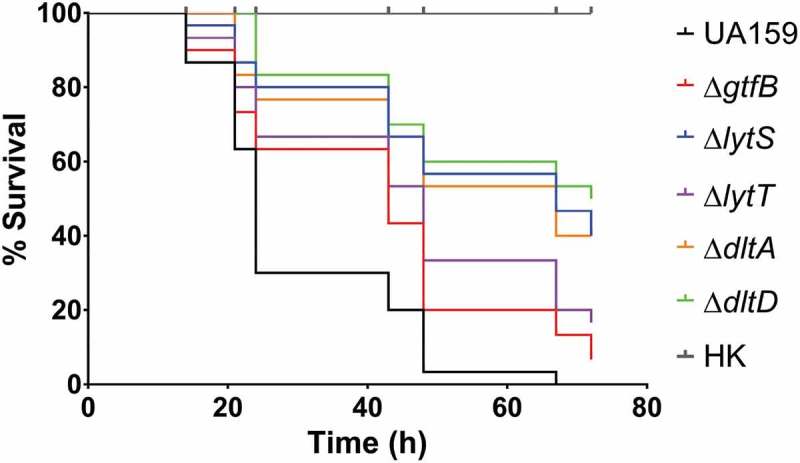
Survival of *G. mellonella* larvae after infection with *S. mutans* parental UA159 and deletion strains Δ*gtfB*, Δ*lytT*, Δ*lytS*, Δ*dltA* and Δ*dltD*. Virulence of *S. mutans* strains displayed by Kaplan-Meier survival curves. Estimates of differences in survival were compared using the Matel-Cox Log-rank test. All larvae survived the heat-killed (HK) control of all strains tested, therefore, the data shown for HK are representative results from one strain.

The deletion strains were then tested in an *in vivo* model of infection, *G. mellonella*. Using similar inocula (Figure S2), all the deletion strains were able to survive longer in the larvae than the parent strain, UA159, which killed all larvae after 67 h of infection (Figure 4; Table S2). In addition, only 7% of the larvae inoculated with ∆*gtfB* survived at 72 h, demonstrating higher systemic virulence potential of this strain when compared to the other deletion strains. Specifically, the larvae inoculated with ∆*lytT* (16.6%), ∆*lytS*, (40%), ∆*dltA* (40%) and ∆*dltD* (50%) showed a higher percentage of survival at 72 h after inoculation. Thus, the ∆*dltD* strain was significantly less able to cause larvae killing, compared to the other deletion strains tested (Figure 4). These results indicate that the products of the genes examined in this study may play a role in influencing how *S. mutans* thrives in systemic infections.

### S. mutans strains’ tolerance to hydrogen peroxide

**Figure 5.**
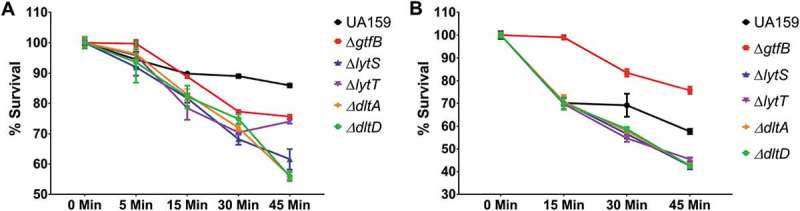
*S. mutans* parental UA159 and deletion strains Δ*gtfB*, Δ*lytT*, Δ*lytS*, Δ*dltA* and Δ*dltD* tolerance to hydrogen peroxide. The graphs show the different survival percentages of the strains population from planktonic culture (a) and biofilms (b) after longitudinal exposure to H_2_O_2_. The data shown are the means, and the error bars correspond to the standard deviation (n = 6 per group).

To further examine the mechanism by which the gene deletion strains were able to survive in the *G. mellonella* larvae, the ability of the strains, grown in planktonic or biofilm cultures, to survive exposure to H_2_O_2_ was determined. In planktonic cultures, the parent strain exhibited the greatest ability to survive a peroxide challenge following 45 min of exposure. There was a gradual decrease in the survival percentage of *S. mutans* in planktonic cultures (Figure 5(a)) and biofilms (Figure 5(b)) of ∆*lytT*, ∆*lytS*, ∆*dltA*, and ∆*dltD* strains after exposure to H_2_O_2_, except for the planktonic culture of ∆*lytT* that increased its population when it passed from 30 to 45 min. However, the *∆gtfB* strain was significantly better able to withstand a peroxide challenge when grown in a biofilm culture. This finding suggests that a decreased amount of insoluble exopolysaccharides found in the extracellular matrix of *∆gtfB* biofilm cultures [7] did not facilitate H_2_O_2_ killing, and that other factor(s) may be playing a role (*e.g*., cell membrane composition and turnover). The ability of the *∆gtfB* strain to persist in biofilm cultures exposed to H_2_O_2_ is borne out in the results observed in our *in vivo G. mellonella* infection model (Figure 4).

## Discussion

The present study demonstrated that the *lytST* and *dltAD* genes affect *S. mutans* virulence in a rodent model of caries and a systemic infection assay. All rats infected with the *S. mutans* parental strain UA159 and deletion strains developed carious lesions. Greater numbers of enamel and dentinal lesions were measured in teeth of animals infected with the parental UA159 strain on the smooth and sulcal surfaces of the animals’ teeth. However, in the animals infected with the deletion strains Δ*gtfB*, Δ*lytS* and Δ*dltD*, caries lesions were less severe; moreover, these deletion strains did not cause severe dentinal caries. Thus, the absence of the products encoded by *gtfB, lytS* and *dltD* resulted in decreased cariogenicity of *S. mutans*. In addition, loss of the *gtfB, lytS, lytT, dltA* and *dltD* gene products prevented the killing of *G. mellonella* larvae after systemic infection with *S. mutans* (compared to the UA159 parental strain), indicating that these five gene products may also influence systemic infections, by reducing the virulence of the organism. This reduction in systemic infection may be in part due to the absence of these genes made the deletion strains (except Δ*gtfB*) more susceptible to oxidative stress, which is part of the innate immune system of *G. mellonella*.

The two *in vivo* models were selected with distinct purposes; the rodent model was to assess the cariogenic potential of strains with deletion of specific genes, and the invertebrate model as an incipient model to evaluate whether the deleted genes could impair the systemic infection potential of *S. mutans*. Specifically, because the deletion strains caused fewer caries lesions with lower severity (vs. the parental strain), the specific genes (and their products) could be targets for directed preventive/therapeutic approaches. Thus, if such approaches would be implemented, there was no information whether targeting these genes would interfere with *S. mutans* behavior in case of bacteremia (e.g., once it goes to the host bloodstream during oral hygiene procedures like flossing). There was no information whether the attenuated virulence phenotype to cause caries would also present an attenuated phenotype in a systemic infection. Therefore, a simple systemic model (larvae) was selected to verify how the deletion strains would affect *S. mutans* virulence.

In the caries model, the microbial population recovered from rats showed a higher proportion of *S. mutans* in the total microbiota for UA159 vs. deletion strains (15-fold for Δ*gtfB*, 3-fold for ∆*lytS* and 6-fold for ∆*dltD*). Recently, a library of UA159 transposon mutants was inoculated in mice to identify genes that are important for colonization and survival in the oral cavity; after 3 weeks, the mutant strains *gtfB, dltC, dltD* and *lytT* presented lower recovery, while *dltA, dltB* and *lytS* were recovered at a similar rate as UA159, but no caries status was reported [41]. Here, the deletion strains ∆*dltA* and ∆*dltD* growth in TY+1% glucose were slower compared to the parental strain UA159 and the deletion strains ∆*gtfB*, ∆*lytS*, and ∆*lytT*. These two strains took 40 to 60 min more to reach mid-exponential (O.D._540nm_ ~ 0.5) and late exponential growth phase (O.D._540nm_ ~ 1.0). Thus, only ∆*dltA* and ∆*dltD* presented a slow-growth defect that may explain in part the outcome for lower colonization in the rats’ teeth and fewer and less severe carious lesions observed. Initial work with ∆*lytT* (∆*lytR*) grown in Brain Heart Infusion broth demonstrated a defect in cell division leading to longer chains because of defects in autolysin activity but without changes in planktonic growth rate or biofilm formation [25]. Later, in a comprehensive study of a genomic collection of gene deletion mutants, the strains were categorized into several growth traits [26]. Both Δ*dltA* and Δ*dltD* had poor acid survival in planktonic cultures and single-species biofilm formation deficiencies on polystyrene surfaces in the presence of sucrose or glucose [26]. The deficiency in biofilm formation on hydroxyapatite was not observed in our *in vitro* study, which used TY culture media with saliva and sucrose alternated with sucrose plus starch [8]. Furthermore, the pH of spent culture media did not differ between strains grown in single-species biofilms, indicating that all strains grew as the pH of culture media dropped from 7.0 to ~4.5 [8]. Nevertheless, the slower growth and lower acid tolerance of Δ*dltA* and Δ*dltD* may contribute to the lower percentage of *S. mutans* recovered and fewer (and less severe) caries lesions.

In the current study, for smooth surfaces, UA159 yielded ≥50% more lesions than the deletion strains on enamel, while it caused the highest amount of caries lesions in dentin (≥80% more cavities). For sulcal surfaces, UA159 yielded ≅ 30% more lesions than the deletion strains on enamel, while UA159 also caused a higher amount of caries lesions in dentin (being ≥60% more severe than the deletion strains). *S. mutans* possesses several virulence factors associated with cariogenicity such as adhesion capacity, acidogenicity, acid tolerance, production of bacteriocins (mutacins) and biofilm formation [10,42]. However, the ability to synthesize water-insoluble exopolysaccharides is a trait that makes *S. mutans* a significant pathogen of dental caries [2]. The synthesis of exopolysaccharides depends on the expression of Gtfs by this microorganism in the presence of dietary carbohydrates [9]. Lack of working *gtfB* was related to low levels of carious lesions on the smooth surfaces of rat teeth [28,43]. Thus, it was expected that the *ΔgtfB* strain would cause less carious lesions on the surfaces of the teeth in the present study.

The infection by Δ*lytS* caused fewer caries lesions in enamel and dentin of rats than the parental strain UA159. Thus, the interruption of the two-component system *lytST* affected *S. mutans* cariogenicity. The *lytST* complex is essential for *IrgAB* expression in *S. mutans*, which participates in oxidative stress tolerance, biofilm build-up and regulation of autolysis [44,45]. The eDNA released by autolysis interacts with GtfB and LTA and affects the production of exopolysaccharides, colonization by *S. mutans*, and construction of the extracellular matrix [8,12,17]. Previously, our group described an increased amount of eDNA in the matrix of *in vitro* biofilms formed by Δ*lytS* and Δ*lytT* [vs. the parental strain; 8]. We proposed that the loss of these genes was affecting *S. mutans* cell metabolism by increasing cell wall turnover, thereby increasing the secretion of microvesicles containing eDNA [17], or triggering additional pathways that culminate in the augmented release of DNA into the matrix [8]. The induction of additional pathways could explain the decreased cariogenicity of Δ*lytS in vivo* observed in this study.

In addition, the *ΔdltD* strain also showed a lower number of caries lesions in enamel and dentin of rats (vs. the parental strain UA159), suggesting that the product of the *dltD* gene could also be involved in the cariogenicity of *S. mutans*. This finding was expected since the *dltABCD* operon genes are required for the addition of D-alanine residues during LTA synthesis [19]; these residues affect bacterial adhesion to the surface and formation of biofilms [22,23,46,47]. Also, a previous *in vivo* study induced the expression of the *dltABCD* operon and demonstrated a higher occurrence of caries [48]. Furthermore, the *S. mutans* proteins encoded by the *dltA, dltC* and *dltD* genes were abundant during the early stages of cariogenic biofilm formation and were related to the amounts of intracellular polysaccharides [15,23]. Therefore, the loss of genes related to the synthesis of exopolysaccharides, eDNA and LTA metabolism impair cariogenicity of *S. mutans*.

In an earlier *in vitro* study, it was expected that the deletion of *lytS* and *lytT* would decrease the amount of eDNA, and deletion of *dltA* and *dltD* would reduce the quantity of LTA in the matrix (vs. the parental strain). However, the data showed more eDNA and LTA in biofilms with the respective deletion strains and that the 3D structure was distinct [8]. Based on these data, it was expected that the strains yielding more eDNA and LTA in the matrix would cause more caries lesions; but this was not the outcome in the rodent model of dental caries here. An explanation is that the deletion of *lytST* and *dltAD* alters *S. mutans* physiology to form biofilms with distinct quantities of extracellular matrix components *in vitro* [8]. These traits could be because of alterations in the cell wall turnover and composition (and charge), as suggested previously [13,44,45,49,50]. The cell wall works as an ‘anchor’ for matrix components to connect to cells surface and mediates cell-cell binding, cell-matrix, cell-matrix-dental surface. Hence, alterations in this ‘anchor’ could lead to a weaker, smaller (with fewer cells connected to each other and to the matrix; that is, lower percentage of *S. mutans* recovered from rats’ dental biofilms) and more ‘porous’ 3D structure that would retain less acid inside the biofilm and at the interface biofilm/dental surface, causing fewer caries lesions. Thus, a more ‘porous’ 3D structure might be related to the distinct patterns of the distribution of bacteria and exopolysaccharides observed in the *in vitro* biofilms (vs. parental strain UA159), represented as percentage coverage per area from the interface substratum/biofilm (hydroxyapatite disk) to the top (outer layer) of each biofilm [8].

Oral bacteria can enter the bloodstream, and usually, bacteremia can be rapidly eliminated by the immune system. However, it has been shown that if *S. mutans* persists in the bloodstream, it can colonize the endothelial cells of the coronary arteries [36]; the cerebral arterial wall [51], the renal arterial wall [52], and even reach the hepatic veins in the liver [53]. *G. mellonella* has a pathogenic defense mechanism similar to the innate immune system of mammals [54], and their hemolymph is considered an analog to blood in vertebrates. This invertebrate model was used to analyze *S. mutans* pathogenesis in systemic diseases such as bacterial endocarditis [35–37]. Here, the deletion of the *gtfB, lytS, lytT, dltA*, and *dltD* genes caused lower and slower larval mortality rates. However, the Δ*gtfB* strain had a higher capacity to kill more larvae when compared to the other deletion strains. The larvae defense mechanisms include a plasmacyte-mediated melanocyte process [38], and the production of reactive oxygen species (ROS), enzymes, and antimicrobial peptides [40]. But, as *G. mellonella* is a limited, preliminary systemic model, further studies will need to be performed to address the contribution of these genes to *S. mutans* systemic virulence. In addition, the mechanism by which *S. mutans* reacts to the stress caused by the immune system of *G. mellonella* is still to be elucidated.

Nevertheless, *S. mutans* is susceptible to oxidative stress [55]. Here, the Δ*lytS*, Δ*lytT*, Δ*dltA* and Δ*dltD* strains revealed lower tolerance to H_2_O_2_, making these genes important for providing resistance to oxidative stress. The Δ*gtfB* strain showed greater survival to H_2_O_2_ exposure than the other deletion strains, possibly due to the presence of *lytST* and *dltAD* that could together alleviate the deleterious effects of hydrogen peroxide. The lack of a protective matrix in *ΔgtfB* could render this strain more susceptible to oxidative challenge, but the data showed an opposite outcome. Thus, we hypothesize that the absence of a working *gtfB* gene changes the cell wall/membrane making *S. mutans* cells more tolerant to oxidative stress when grown in a biofilm. Further research is needed to pinpoint the exact mechanism for ∆*gtfB* higher tolerance to H_2_O_2_ and explain why it killed more larvae than the other four deletion strains tested. For example, transcriptomic and/or proteomic analyses of ∆*gtfB* challenged with H_2_O_2_ could indicate the mechanisms involved in the observed tolerance. Nevertheless, previous gene expression analysis indicated that *ΔgtfB* might present a slower turnover in the cell membrane in mixed-species biofilms [13, used the same strain *ΔgtfB* used here], corroborating with earlier work using a *ΔgtfBC* strain [56].

Moreover, bacterial adaptation to changing environmental conditions is often accomplished by TCSs that modulate gene expression in response to many stimuli [57]. A previous study showed that the Cid/Lrg systems interact together to favor the colonization of *S. mutans* against environmental stresses [58]. The expression of *IrgAB* is dependent on the *lytST* genes and confers resistance to oxidative stress [44,45]. LytST (*lytS*: sensor kinase; *lytT*: response regulator) is required to activate the expression of *lrgAB*, which is part of the *S. mutans* arsenal that controls biofilm formation and autolysis [45]. The *S. mutans IrgAB/lytST* deletion strains exhibited a decreased growth rate in the presence of several ROS [44,45]. Before, the expression pattern was distinct for *lytS* and *lytT*. The expression of *lytS* was influenced by pH and abundance of carbohydrates, while *lytT* was expressed in very low magnitudes at distinct developmental stages for both UA159 and Δ*gtfB* in mixed-species biofilms, regardless of environmental pH or nutrient availability [13]. The similarity between both types of biofilms indicates that *lytT* may interfere with multiple metabolic pathways simultaneously.

Furthermore, as the Δ*gtfB* biofilm aged, the expression of *lytS* decreased, being inversely proportional to the increase of expression recorded for *lrgAB*, which was not observed for UA159 biofilms. Even though LytST controls the expression of *lrgAB*, a Δ*lrgAB* mutant presented a distinct transcriptomic profile from the Δ*lytS* mutant [45], suggesting that changes in gene expression by the mutation of *lrgAB* could be independent of LytST [59]. Further comparison of transcriptomics and proteomics of a Δ*lrgAB* mutant (vs. the parental strain) subjected to distinct oxidative, heat and antimicrobial stresses indicated ‘that adaptation to a new environment may require radical proteome turnover or metabolic remodeling’ [50]. Among the pathways (and resulting products) were several genes/proteins involved in cell wall biogenesis affecting cell wall turnover and stress tolerance (e.g., GtfC, AtlA, DltC) [50]. In addition, alteration in the cell wall of *dlt* mutants may also occur.

The deletion strains ∆*dltA* and ∆*dltD* exhibited the same behaviors as the ∆*lytS* and ∆*lytT* when inoculated in *G. mellonella* larvae. Thus, these genes could also be contributing to the virulence of *S. mutans* in systemic diseases and cooperation for the tolerance to ROS products. Moreover, the expression of the *dlt* genes was related to the resistance of *S. mutans* biofilms against gentamicin, a drug used to treat endocarditis [49]. The *ΔdltA* showed a more negatively charged surface than the parental strain, reducing the tolerance of the *dltA* mutant biofilms towards the positively charged gentamicin [49]. Furthermore, it was shown that *dltABCD* affects the susceptibility of *S. mutans* to cationic antimicrobial peptides (innate immune factors in humans) [60]. It has been shown that a D-Ala analog can block the activity of DltA and synergize with the peptidoglycan synthesis inhibitor vancomycin, leading to growth inhibition [61]. Additionally, a small molecule was identified as a LtaS enzyme inhibitor [62] that inhibits the growth of several medically critical Gram-positive bacteria [62,63]. Our preliminary work with this small molecule has shown to inhibit *S. mutans* growth in planktonic culture and biofilms (Castillo Pedraza et al., unpublished data). Therefore, the higher survival of larvae infected with Δ*lytS*, Δ*lytT*, Δ*dltA*, and Δ*dltD* may be related to the strains’ lower tolerance to oxidative stress after exposure to H_2_O_2_, and host antimicrobial peptides [60]. Thus, it is possible that changes on cell wall/membrane in the deletion mutant tested here could explain some of the distinct and even unexpected outcomes, and thus, future research on cell wall/membrane composition and structure is warranted.

In conclusion, besides the roles that the *lytST* and *dltAD* genes play in the development and composition of *in vitro* biofilms [8], the results from this study demonstrate that they are also factors in the virulence of *S. mutans* in the development of dental caries and systemic infection. Inhibition of these genes and their products with specific therapies would help decrease the virulence of *S. mutans* to prevent the deleterious effects of demineralization and reduce microorganism survival in systemic infections.
