# Supplementary material for: Inactivation of Streptococcus mutans genes lytST and dltAD impairs its pathogenicity in vivo
Source: J Oral Microbiol. 2019 May 9;11(1):1607505. doi: 10.1080/20002297.2019.1607505 (PMC6522913; doi:10.1080/20002297.2019.1607505)
Supplement: Supplemental Material [file ZJOM_A_1607505_SM1266.docx]

**SUPPLEMENTAL MATERIAL**

**Table S1.** Diet 2000.

| Table S1. Contents of 2000 Diet (%)* |
| --- |
| 1% desiccated liver (DIFCO)  2% iodized salt (Cisne)  1% of protein in Alfalfa (20.29% protein-Accorsi Medicinal Plants)  5% of yeast powder extract (DIFCO)  7% of whole wheat flour (Renata)  28% of skimmed milk powder (La Serenissima)  56% of powdered icing sugar (Malvalério) |

*The dietary material throughout the experiment came from the same manufacturing batch (Bowen *et al*., 1988).

**
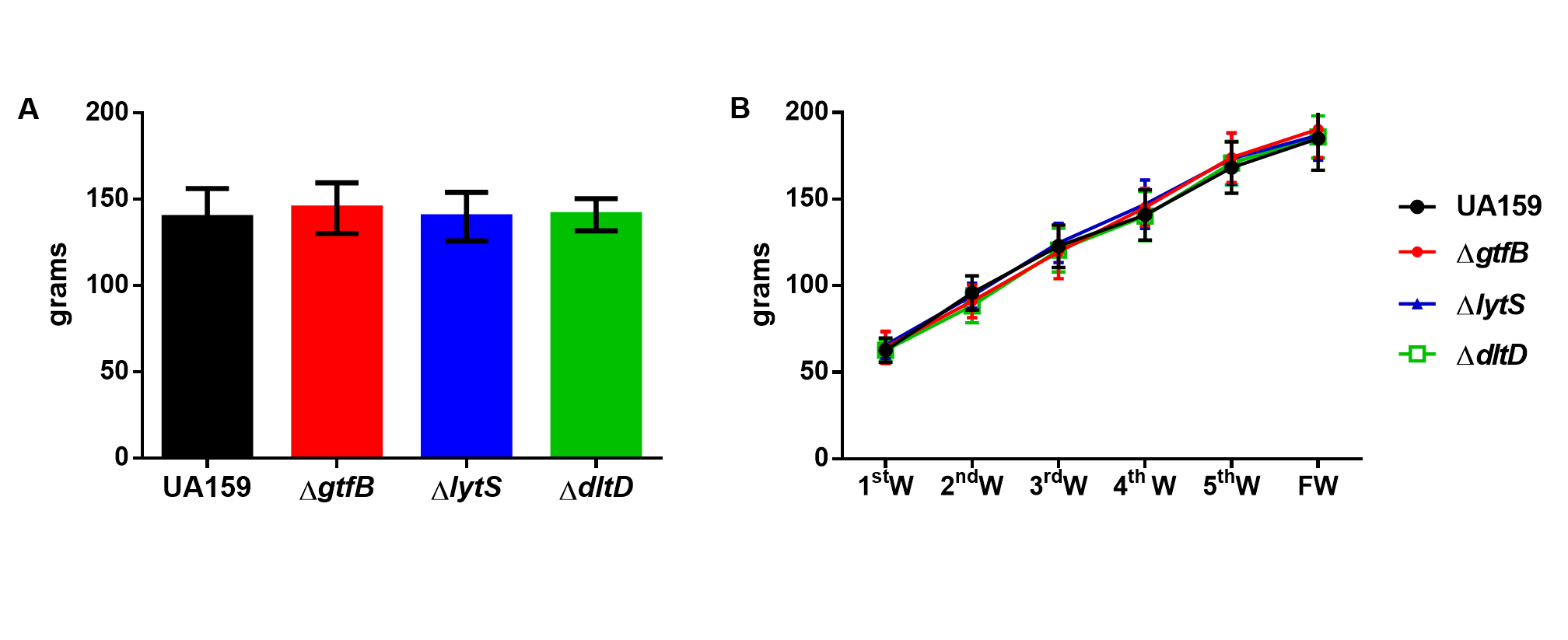
**

**Figure S1.** The weight of rats during the dental caries experiment. (**A**) The overall weight was similar between the different groups (p > 0.05, one-way ANOVA). The data represented are the means, and the error bars correspond to the standard deviation (n = 14 per group). (**B**) Longitudinal growth of rats. A significant increase in the weight of all animals occurred within each group over time (p < 0.0001 for each strain, two-way ANOVA and repeated measures, followed by Tukey's test). Thus, the Diet 2000 and other care during the maintenance period allowed all rats to grow equally well, as expected. These circumstances may provide an ideal environment for the implantation of microorganisms tested in the oral cavity.

**Figure S2.** Quantity of *S. mutans* parental UA159 and deletion strains *ΔgtfB*, *ΔlytT*, *ΔlytS*, *ΔdltA* and *ΔdltD* inoculated into the hemocoel of *G. mellonella* larvae in the three experiments performed. The data represented are the means, and the error bars correspond to the standard deviation.

Table S2. Summary of *G. mellonella* larvae percentage survival over time.

| Time (h) | Strains | | | | | | |
| --- | --- | --- | --- | --- | --- | --- | --- |
|  | UA159 | *ΔgtfB* | *ΔlytS* | *ΔlytT* | *ΔdltA* | *ΔdltD* | HK |
| 0 | 100.00 | 100.00 | 100.00 | 100.00 | 100.00 | 100.00 | 100.00 |
| 14 | 86.6 | 90.0 | 96.6 | 93.3 | 100.0 | 100.0 | 100.00 |
| 21 | 63.3 | 73.3 | 86.6 | 80.0 | 83.3 | 100.0 | 100.00 |
| 24 | 30.0 | 63.3 | 80.0 | 66.6 | 76.6 | 83.3 | 100.00 |
| 43 | 20.0 | 43.3 | 66.6 | 53.3 | 66.6 | 70.0 | 100.00 |
| 48 | 3.3 | 20.0 | 56.6 | 33.3 | 53.3 | 60.0 | 100.00 |
| 67 | 0.0 | 13.3 | 46.6 | 20.0 | 40.0 | 53.3 | 100.00 |
| 72 |  | 7.0 | 40.0 | 16.6 | 40.0 | 50.0 | 100.00 |

All larvae survived the heat-killed (HK) control of all strains tested, therefore, the data shown for HK are representative results from one strain.
